# Perceived impact of a homeless shelter-based art program on stress, social connection, and emotional regulation: a mixed-methods pilot study

**DOI:** 10.3389/fpsyg.2026.1825416

**Published:** 2026-08-26

**Authors:** Mian Horvath, Sadik Karma, Riley Theriault, Luis Antonio Velez Figueroa, Rocio Chang

**Affiliations:** University of Connecticut Health, Farmington, NM, CT, United States

**Keywords:** art creation, community, coping skills, de-stress, emotional regulation, homeless shelter

## Abstract

Individuals experiencing homelessness often experience chronically high levels of stress that adds an additional challenge to their lives. Barriers such as stigma, distrust, and accessibility limit engagement with traditional mental health services. Art creation may offer an accessible, non-clinical complement to existing supports by creating space for emotional expression, reflection, and self-directed coping. This pilot study examined the feasibility, acceptability, and perceived impact of an art creation program for guests of a homeless shelter. Utilizing a mixed-methods approach, the program effects were evaluated based on 1) observations from session facilitators, 2) semi-structured interviews with shelter staff and leadership, and 3) pre and post-session participant surveys. Thematic analysis of facilitator reflections identified three primary themes: (1) art as a welcomed respite from institutional monotony, (2) structured art creation as a space for self-reflection and emotional expression, and (3) art as a catalyst for social connection. The third theme included subthemes highlighting how art-centered conversations fostered connection through shared experiences and supported vulnerability. Shelter staff reported increased spontaneous use of art materials outside structured sessions and perceived reductions in interpersonal conflict, suggesting broader cultural integration of art as a coping tool. Participant surveys demonstrated repeat participation, reduction in stress, and that many participants utilized skills gained during the sessions in their everyday life. This pilot study suggests that simple art creation sessions are well received by shelter guests and are associated with perceived stress reduction and increased usage of art as a coping strategy. Art-based interventions may represent a scalable, low-cost approach to complement existing behavioral health supports in shelter-based settings.

## Background

People experiencing homelessness face complex challenges extending beyond readily apparent physical and economic challenges including structural violence, stigma, and substance/alcohol use disorder (Fazel et al., 2008; Milaney et al., 2019; Reilly et al., 2022). These intersecting stressors contribute to chronically high levels of stress. Prolonged stress exposure is associated with worsening of behavioral health and emotional dysregulation (Cleck and Blendy, 2008; Schneiderman et al., 2005). Among individuals experiencing homelessness, persistent stress and limited access to emotional regulation resources may reduce engagement with available supports and create additional barriers to stable housing, thereby increasing the risk of ongoing or recurrent homelessness (Piat et al., 2015; Zhao, 2023). Therefore, accessible and scalable strategies to promote stress management and emotional regulation among people experiencing homelessness are critically needed.

Although many homeless shelters offer access to formal behavioral health services, these services are often underutilized (Moon et al., 2024; Thorndike et al., 2022; Winiarski et al., 2020). Barriers to engagement include limited accessibility, perceived stigma, and institutional distrust (Kerman et al., 2019; Thorndike et al., 2022). Even when available, traditional therapeutic services may not be feasible for all shelters due to financial constraints. These limitations highlight the need for complementary, resource-efficient interventions that can be embedded directly within homeless shelters.

Art therapy is a psychotherapeutic approach that integrates creative expression with structured clinical processing and has benefits across a range of mental health conditions including depression, anxiety, cognitive impairment, schizophrenia, self-esteem, trauma, and social adjustment (Bosman et al., 2021; Hu et al., 2021; Joschko et al., 2024; Quinn et al., 2025). However, art therapy is delivered by a licensed art therapist, requiring specialized graduate training with appropriate credentialing. As of 2021, the Art Therapy Credentials Board (the licensing organization for art therapists) reported 4,550 active credentialed art therapists within the United States of America (ATCB, 2021). Given the limited quantity of art therapists and institutional financial limitations, licensed art therapists are often inaccessible in community-based or shelter settings (Regev and Cohen-Yatziv, 2018). As a result, most programs for individuals experiencing homelessness are unable to implement formal art therapy despite its potential benefits. While there are some studies demonstrating art therapy programs for children experiencing homelessness can decrease depressive symptoms, support more adaptive responses to stress, and help children express challenging emotions (McLaughlin, 1990; Woollett et al., 2020), there is limited literature regarding the role or effect of art therapy for adults experiencing homelessness, likely in part due to limited accessibility of art therapy. This gap raises an important question: how can creative interventions be delivered in settings where licensed art therapy is not available?

Beyond formal art therapy, emerging evidence suggests that art creation itself, independent of structured psychotherapy or a licensed art therapist, may support mental health (Beauchet et al., 2022; Bungay et al., 2023; Schwan et al., 2018). Understanding *why* art creation may reduce stress requires attention to both its psychological and social mechanisms. At the most immediate level, the act of making art appears to have a direct physiological effect with reductions in salivary cortisol after 45 min of art making, regardless of prior artistic experience (Kaimal et al., 2016). This finding suggests that the stress-reducing properties of art creation are not contingent on skill, but rather emerge from something more fundamental about the act of creative engagement itself. From a psychological standpoint, one candidate mechanism is flow, or a state of focus in which attention narrows away from external stressors, self-consciousness recedes, and a sense of agency emerges (Chilton, 2013). This quality of focus may be especially meaningful in shelter environments, where chronic stress and constrained daily routines leave little room for self-directed engagement.

Art creation also provides a medium for externalizing and processing emotional states that are difficult to articulate verbally, supporting meaning-making and a sense of personal agency in ways that purely conversational approaches may not (Spandler et al., 2007). For individuals experiencing homelessness, arts-based programming has been described as a pathway to healing, empowerment, and the reclamation of voice and identity, outcomes that extend well beyond momentary stress relief (Murphy and Alexander, 2020).

The context in which art making occurs also shapes its impact. Allen (1995) introduced the open studio as a non-clinical, non-hierarchical creative space in which participants and facilitators engage as co-participants rather than in a therapeutic hierarchy, and Timm-Bottos (2017) extended this model through community art studios that function as accessible third spaces fostering social connection and community-defined wellbeing. A review by Finkel and Bat Or (2020) posited that such open studio models consistently produced benefits such as reduced stress, improved mood, increased self-efficacy, and strengthened social belonging across diverse community settings, outcomes that speak directly to the unmet needs of individuals residing in homeless shelters.

Relating to individuals experiencing homelessness, two theoretical frameworks inform our understanding of how art creation may benefit them. Self-Determination Theory (SDT; Deci and Ryan, 2000) proposes that psychological wellbeing depends on the satisfaction of three basic needs: autonomy, competence, and relatedness. Homeless shelter environments, though not their goal, often constrain all three. Residents have limited agency over daily routines, few opportunities for skill development, and may experience social isolation despite shared physical space. Programs that prioritize participant choice, skill-building, and group-based engagement may address these unmet needs directly. Additionally, Fredrickson (2001) Broaden-and-Build Theory proposes that positive emotional states, such as enjoyment, playfulness, and a sense of connection, expand the range of thoughts and actions available to a person in the moment. Rather than narrowing attention toward immediate threats or stressors, as negative emotions tend to do, positive emotions open space for curiosity, creativity, and social engagement. Critically, these momentary expansions are not fleeting, i.e., with repeated experience, they accumulate into lasting personal resources: stronger relationships, greater resilience, and a broader repertoire of coping strategies. For individuals experiencing homelessness, whose daily environments are often characterized by chronic stress and constrained choices, even brief but consistent opportunities for positive emotional experience may carry disproportionate benefit. Structured art creation sessions (recurring, low-pressure, and socially engaging) may represent precisely this kind of opportunity.

To further explore the potential benefits of art creation in the context of a homeless shelter, outside of the theoretical, we implemented a structured, informal, resource-conscientious art creation pilot program within a congregate homeless shelter in the United States of America. We hypothesized that embedding recurring, low-pressure, autonomy-supportive art creation sessions within the shelter would support perceived stress reduction, create opportunities for self-expression and social connection, and allow participants to practice, strengthen, or adapt coping strategies that could be used beyond the sessions.

## Methods

### Study design

This study used an exploratory mixed-methods design to evaluate the implementation and participant experiences of an art creation program within an urban homeless shelter in the United States of America. Data were collected from session facilitator observations and experiences, semi-structured interviews with shelter staff and leadership, and pre and post-session surveys. This study was performed under IRB #25X-333-1 exemption as determined by University of Connecticut Health. Because this was a pilot study, we prioritized feasibility, acceptability, implementation, and perceived impact rather than hypothesis testing or definitive efficacy evaluation (Bowen et al., 2009; Orsmond and Cohn, 2015).

### Setting and program description

The program was developed collaboratively with leadership at a single urban co-ed homeless shelter housing individual adults (no family units) in the United States of America. The sessions were not structured psychotherapy and did not include formal therapeutic processing or clinical assessment.

It is important to note that the program was intentionally designed as an art *creation* intervention rather than art *therapy*, and this distinction was central to the study’s aims. Art therapy requires delivery by a licensed art therapist, which many shelter-based programs cannot realistically access. By designing a program that does not depend on licensed art therapists, we sought to develop a model that is replicable in resource-limited community settings. Additionally, as discussed in the introduction, art creation, or open studio, has therapeutic benefit fostering social connection and community wellbeing, improving mood, and encouraging self-efficacy.

Each session was overseen by a licensed clinical psychologist and facilitated by trainees. The psychologist’s role was not to conduct therapy, but to ensure participant safety and to provide trained support. Psychologists and trainees participated in art making alongside shelter guests as co-creators rather than as clinicians. This was a deliberate choice to remove the hierarchical dynamic typical of clinical encounters and support relatedness by positioning facilitators and guests as peers, fostering the comfort, trust, and openness that the program depended on.

All shelter guests were invited to attend but attendance was not mandatory. Shelter staff were also invited to participate during their downtime. Initially, sessions were offered once monthly; due to participant interest, frequency increased to twice monthly. Each session lasted approximately 1 hour.

Sessions were held in the shelter’s dining room, a large communal space with round tables that allowed small-group interactions while remaining open to the broader shelter environment. Each session began with the licensed clinical psychologist introducing themselves by first name, without their title, followed by an overview of that session’s art mediums and reflective prompt. Prompts were developed collaboratively by the facilitator team prior to each session and were designed to encourage reflection and self-expression. Example prompts included: What do you love about your body; What part of your heritage makes you feel the proudest; and What is something you worry about that you are scared to share with others. Prompts were written on placards at each table. The bulk of the session was left open for participant-determined art creation. Conversation was encouraged at each table but left to flow in whatever direction it took. At the end of each session, participants were offered space to share their thoughts, reflections, or art if they wished. Informal feedback gathered during sessions was used to inform and refine subsequent prompt development.

At each session, we offered multiple visual art materials such as coloring pages, free drawing with colored pencils, chalk, pastels, and blow painting. Other sensory activities included essential oils and a Tibetan singing bowl. Participants were free to choose their preferred medium and sometimes moved between mediums throughout the session. By giving participants freedom to choose their medium, we hoped to foster a greater sense of autonomy among the shelter guests. Due to the varied art options, creative output varied from coloring and doodles to abstract art and highly detailed, precise drawings. Participants were free to join and leave at will throughout the session. While some did come and go, the majority stayed for the whole session, creating art and conversing with their table.

The program was designed around the three guiding principles: accessibility, informality, and participant autonomy.

#### Accessibility

Sessions were held onsite to reduce transportation and scheduling barriers. Organizing the program within the shelter allowed guests to attend without additional appointments or referrals. Sessions were advertised by flyers and were held the same time each month to encourage routine. Moreover, we hoped that guests would view the shelter as a safe space, one where they could feel more comfortable and willing to be vulnerable than they might be in a healthcare office.

#### Informality

Facilitators were distributed across tables and participated in art making alongside shelter guests to reduce hierarchical dynamics and increase guest comfort. When sharing reflections, facilitators were encouraged to model participation to foster a norm of mutual engagement.

#### Participant autonomy

Multiple visual arts were available at each session, and participants were free to select and change mediums throughout the session. This flexibility was intended to support autonomy as it has been shown to increase utilization of healthcare resources by people experiencing homelessness (Magwood et al., 2019) and encourage psychological wellness.

### Participants

Study participants included shelter guests, shelter staff and leadership, and session facilitators. Shelter guests included adults who voluntarily attended one or more art session during the six-month study period. Attendance was open and no exclusion criteria were applied. Because sessions were open, attendance varied by session but typically ranged 5–15 shelter guests. Half of the guest respondents had ages ranging from 26–45 and only one respondent was younger than 25. Thirteen guest respondents identified as female and seven as male.

Shelter staff members included those who worked second shift while the sessions occurred. There were approximately 5 staff members who worked second shift on the days the art sessions occurred over the six-month period. Shelter leadership included those who were physically onsite and regularly interacted with guests. Demographics were not collected from shelter staff and leadership to help maintain anonymity.

Session facilitators included the supervising clinical psychologist and facilitating trainees. Of the facilitators who responded to the survey, four identified as female, two as male, and two as non-binary. Additionally, almost all had ages ranging from 18–35.

### Data collection

Data was collected in three ways: facilitator recounting, shelter staff and leadership interviews, and pre and post-survey interviews. This multi-tiered mixed-methods analysis allowed us to obtain perspectives of all parties involved with the sessions. A mixed-methods approach, grounded in pragmatism, suited our research questions. It allowed us to draw on the strengths of both quantitative and qualitative methods, producing a more holistic picture. Surveys provided a quantitative measurement of participation and effect over time, while facilitator recounting and staff interviews captured the underlying social complexity (Johnson et al., 2007; Johnson and Onwuegbuzie, 2004). Because this study was based on constructivism and facilitators participated in the sessions alongside guests, their reflections are necessarily situated accounts that shaped the reality observed rather than detached observations (Braun and Clarke, 2019).

Data was collected over the course of 6 months. Reflections were recorded immediately after each session to ground them in concrete events, interactions, and participant statements. *n* = 3 unique facilitators and *n* = 13 reflections total.

Shelter staff interviews were conducted in person with staff while on shift in a private room (*n* = 3 staff). Shelter leadership interviews were conducted virtually (*n* = 1). The interviews were semi-structured and guided by an interview protocol which was developed to explore staff perceptions of the program benefits and their observations of guests who participate in the program (Supplementary Figure S1). All interviews were conducted by the same researcher.

Shelter guests (*n* = 20 unique guests, *n* = 24 total guest surveys) and facilitators (*n* = 7 unique facilitators, *n* = 13 total facilitator surveys) were invited to complete brief anonymous surveys at the beginning and end of each art session. The same survey was used for both participants and facilitators, but facilitators were asked to mark their surveys with an F. The survey included demographics (age range and gender), attendance history, pre and post-survey stress, reason for attending, skills applied outside the sessions, and open-ended feedback on session strengths and recommendations. Stress was reported as a single-item Likert scale: 1–10. The stress reports were a single-item self-report measure intended for brief, session-level assessment. Given the pilot nature of this implementation study and concerns regarding participant compliance, we elected not to administer validated multi-item psychometric stress measurements.

### Analysis

Qualitative data were analyzed using Braun and Clarke’s thematic analysis framework with an inductive, data-driven approach (Braun and Clarke, 2006). The first author familiarized themselves with the data and generated initial codes using line-by-line coding which were then grouped into broader categories to assign initial themes. Consistent with constructivism, facilitators created meaning in collaboration with participants. Therefore, facilitator perspective is inherently a part of meaningful interpretation (Braun and Clarke, 2019). To strengthen the reliability of the analysis, the co-authors individually reviewed the codes and contributed to theme development, ensuring that themes reflected patterns across multiple perspectives rather than any single interpretive lens. Together all authors defined final themes using visual mapping. Themes were reviewed to ensure they had adequate data supporting each of them, specifically that each theme occurred across multiple participants, sessions, and facilitator rather than isolated anecdotes. Themes were also evaluated to ensure coded data was congruent with the theme and that themes were appropriately unique while addressing the research questions. Discrepancies between two authors at any stage of analysis were discussed with all authors until a code/theme could be unanimously agreed upon.

To enable linkage across time while maintaining anonymity, survey respondents were asked to generate a code using the first letter of their first name and the date of their birthday. Some surveys included incomplete or illegible codes, resulting in potentially incomplete linkage. Change in stress and session satisfaction analyses were conducted using an aggregate of all surveys resulting in some respondents having multiple entries (i.e., change in stress levels) due to multiple surveys across different sessions. All other analyses were conducted with only the most recent survey completed by each respondent, to prevent multiple counting of the same participant. Change in stress (*n* = 36 pre and post-survey combinations) was calculated by subtracting the difference in respondent stress between their post-session survey and pre-session survey.

Quantitative data was analyzed using the following R packages: tidyverse (Wickham et al., 2019), paletteer (Hvitfeldt, 2021), scales (Wickham et al., 2025a), and readxl (Wickham, et al., 2025b).

## Results

### Art sessions may encourage community, reflection, and expression

Following each session, facilitators were asked to record their experiences, observations, and conversations with participants. We identified three themes 1) Art can be a welcomed respite from institutional monotony, 2) Structured art creation provides space for self-reflection and self-expression, and 3) Art can serve as a catalyst for social connection. The third theme had the following subthemes 1) Art-centered conversation fosters connection through shared experience and 2) Art-based connections foster vulnerability.

#### Art can be a welcomed respite from institutional monotony

Prior to implementation, shelter leadership expressed skepticism regarding anticipated attendance, noting that participation in optional programs is often low in shelter settings. There were also concerns that art might be perceived as childish, trivial, or only appealing to women. These expectations, however, were not reflected in facilitator observations.

Throughout the duration of the study, participant perception of the program remained very positive. Participants described the sessions as enjoyable and expressed interest in more frequent sessions. Facilitators often observed that people were very happy to create art for the full hour and were disappointed when we packed up and left, asking to be left supplies to continue their art. In fact, repeat attendance was common and facilitators frequently sat at a table with many familiar faces including some participants who had been regulars since the first session. Participants also expressed eagerness to return like one who mentioned that he would like to have more of these sessions and was looking forward to our next one. Another came by to say, “Thank you for coming. I’m not feeling up to attending tonight, but I promise I’ll try to feel up to it next time.”

The most cited reason for enjoyment was that the sessions were a welcome break from an otherwise boring evening in the shelter. For example, one guest said, “I’m normally really bored at the shelter, and this was a nice change of pace” while another said, “This was a nice thing to do to break up the boredom of living in the shelter.” A third shared that although she was only there for snacks, this was good for her, and she planned to attend future sessions as well.

These observations suggest that the sessions were perceived not merely as recreational activities, but as structured interruptions to institutional routine. Within a setting characterized by limited autonomy and repetitive daily structure, the art sessions appeared to offer participants a temporary shift in tempo. The consistency of repeat attendance and spontaneous expressions of appreciation across sessions indicates that this is a shared perception among attendees. These findings suggest that even modest, predictable opportunities for creative engagement may be experienced as meaningful within shelter environments. This highlights the potential significance of structured, voluntary, recreational activities in institutional contexts.

#### Structured art creation provides space for self-reflection and emotional expression

While each session incorporated prompts designed to encourage self-reflection, we were uncertain whether guests would engage in active participation and interactive discussion or independent art making. However, participants engaged actively in both independent art making and shared discussion. Participants frequently created artwork that reflected their personal experiences, values, and identities, which in turn prompted meaningful discussion independent of the prompts. In fact, participant conversations often shaped the development of future prompts.

One topic was heritage. Across different sessions, participants shared how their art represented “Black African American Power,” “Puerto Rican Culture in America,” and pride in speaking a language other than English. In contrast, one woman discussed how she felt she did not have a heritage, taking away from her sense of identity, which she found challenging. Another topic was spirituality. For example, one participant would draw upon God as his source of art inspiration while another discussed that despite life’s challenges, she felt peace knowing that God was looking out for her, which she felt was reflected in her art. A third topic was family. One woman shared how nice it was to be connected to her family and have a grandchild who would check in. She was excited to share her art at the next check in. Another participant used the session to make a title page for her daughter’s journal. A third participant shared how proud she was of her family and was making a drawing that represented all that her family had achieved despite the challenges they had faced. Across these examples, art creation appeared to function as a tangible medium through which participants externalized aspects of their identity that may not otherwise have been welcomed in daily shelter interactions. The program may offer space in which participants felt safe and welcome to share their values and identities with other participants.

The program also created space for participants to process and express their emotional states. One participant described feeling very excited at the idea of getting a job, being able to save up money, and leave the shelter. She had a recent job interview and just had to wait to hear back. The waiting was frustrating, but art helped with the anticipation. This was contrasted by another who expressed frustration at her inability to obtain a job. She had submitted several job applications and had been invited to multiple interviews, but none had resulted in a job. She felt afraid that without a job she would never leave the shelter. Coming to make art was the only thing she looked forward to these days. A third shared that earlier that day she had learned that her dog had been killed rather than dying of natural causes, leaving her very angry and upset. She expressed that art creation provided an outlet for her emotions. More abstractly, one participant noted that since attending these sessions, she now relies on art for the purpose of emotional regulation, as it had helped her tremendously. Another described art as his way of living and expressing himself. A third said that she was really excited for the session because she wanted to paint her feelings on the paper. These examples illustrate the perception among participants that art making offered a contained, socially affirming space for engaging with emotional states that shelter life often leaves little room to process.

Through the program, art creation was consistently, repeatedly used as a vehicle for both identity exploration and emotional processing. Rather than positioning the sessions as producing emotional change, the observations suggest that the sessions were experienced as a space in which reflection, emotional processing, and articulation of these internal processes were possible.

#### Art can serve as a catalyst for social connection

The third theme “Art can serve as a catalyst for social connection” was subdivided into the subthemes: “Art-centered conversation fosters connection through shared experiences” and “Art-based connections foster vulnerability.” Although participants live in the same shelter space, they were often individuals sharing physical space rather than forming a cohesive community. While we anticipated that participants might converse during art making, we were struck by the depth and consistency of connections that emerged organically through the sessions.

Because participants engaged in conversation while creating art, conversations frequently centered on shared values, goals, and lived circumstances, fostering relational bonds. Employment and housing, common stressors within the shelter setting, were frequent topics of discussions. One participant shared that she had secured housing and was moving to it next week, proudly showing pictures of her apartment and describing how she planned to decorate. This prompted another to note that she had applied to an apartment with her boyfriend and was waiting to hear back. The two were delighted to learn that someone else was in a similar situation and continued to share their excitement and suggestions for each other throughout the session. At another session, conversation revolved around job searches, including some participants who were actively applying for jobs, while others were about to start new jobs at FedEx and a daycare. This exchange of experiences flowed bidirectionally with one facilitator sharing that they were anxious about starting a new job. Such moments illustrate how art sessions create opportunities for mutual exchange, enabling participants to celebrate accomplishments and voice uncertainties in a supportive setting.

Participants also connected over shared backgrounds, interests, and identities. Two participants bonded after realizing they were both from the same region in Puerto Rico and spent time reflecting on shared heritage. Another participant connected with two younger residents because he had attended the same high school they were currently attending. Groups also formed around shared interests, such as participants discussing romance novels and drawing inspiration from some of the characters in the books. These interactions suggest that the sessions created opportunities for participants to discover commonalities that might otherwise have not been disclosed with the setting of the shelter. Over time, facilitators observed that individuals who connected in one session often chose to sit together in subsequent sessions, indicating that these interactions were not fleeting but may contribute to sustained bonds.

As trust developed through these shared experiences, participants became more comfortable expressing vulnerability. Support manifested in both physical and emotional forms. Physically, participants assisted one another, such as when one guest could not blow through a straw to push paint, so she worked with someone else to do the paint blowing. More commonly, sessions created space for emotional support and validation. Participants expressed appreciation for being listened to and affirmed; one noted gratitude that the table had listened and validated her anger. Others disclosed personal struggles, including concerns about a friend not receiving adequate mental health care, pride in recent sobriety, and anxiety about upcoming medical appointments and possible need for surgery. One participant described the sessions as “such a light for her in some of her dark times,” highlighting the emotional significance of the program. Importantly, vulnerability did not appear forced by the structured prompts but instead emerged organically through conversation as trust strengthened.

Some sessions were intentionally designed to encourage participants to step outside their comfort zones and experience growth through experimentation, such as creating art using their non-dominant hand or engaging in chair yoga. Although there was initial frustration, participants ultimately expressed enjoyment and playfulness after persevering. The willingness to attempt unfamiliar tasks, and to do so in front of peers, further demonstrated the level of safety and comfort cultivated within the group. During these moments, peers provided verbal encouragement and reassurance, reinforcing collective support.

Overall, the program created space not only for artistic expression but also for relational development, mutual support, and collective growth. Through structured yet flexible artistic engagement, participants moved from co-presence to connection, using art as a shared medium for recognition, mutual support, and belonging. In doing so, art session functioned as a social infrastructure within the shelter, facilitating sustained interactions, strengthening interpersonal trust, and contributing to the emerging community.

### Facilitated sessions may contribute to a culture of art within the shelter

To add depth to the facilitator observations, we conducted interviews of four shelter staff members, including the deputy director (Supplementary Figure S1). Most interviewees had worked at the shelter prior to program implementation, allowing them to comment on perceived changes over time, with duration of employment ranging from 8 months to 3 years.

Staff consistently described the sessions as highly anticipated and well attending, noting sustained participation across seasons, where program attendance usually declines significantly in warmer months when guests can spend time outside. Importantly, interviewees emphasized that such engagement is uncommon for externally facilitated programming, as shelter residents are often understandably cautious with unfamiliar facilitators, given histories of institutional distrust. They attributed acceptance of the program to its informal atmosphere, emphasis on equality, and focus on developing relationships. These characteristics appeared to reduce hierarchical barriers and foster trust, which may explain why participation levels those typically seen only in internally run programs.

Beyond attendance, staff described perceived shifts in how guests used art, interacted with one another, and navigated stressful moments. Interviewees perceived reductions in stress during the sessions and noted that participants appeared calmer afterward. Notably, staff reported guests internalizing art as a coping mechanism. Residents began independently requesting art supplies during periods of emotional distress, a behavior that was new since the introduction of the program. The frequency of these requests ultimately led the shelter to begin supplying art materials outside of formal sessions. This shift suggests that the program may have contributed to embedding art as an emotional outlet within the shelter’s atmosphere. Staff also perceived broader relational shifts, describing what they experienced as a calmer social environment and attributing this partly to guests’ use of art during emotionally heightened moments. Staff perceptions indicate that the program may have influenced the social climate of the shelter.

Collectively, staff perspectives suggest that the facilitated sessions extended beyond isolated programing and may influence daily practices and norms. Art creation offers a constructive outlet in an environment with limited structured activities. Rather than functioning solely as a distraction, the sessions were perceived to provide the participants with a strategy for emotional coping. The emergence of art making independently of the sessions and integration of art supplies into the shelter environment may reflect the development of a sustained “culture of art,” one in which creative expression became both socially accepted and used as a coping tool.

### Participants reported reduced perceived stress and use of art-related coping strategies

**Figure 1:**
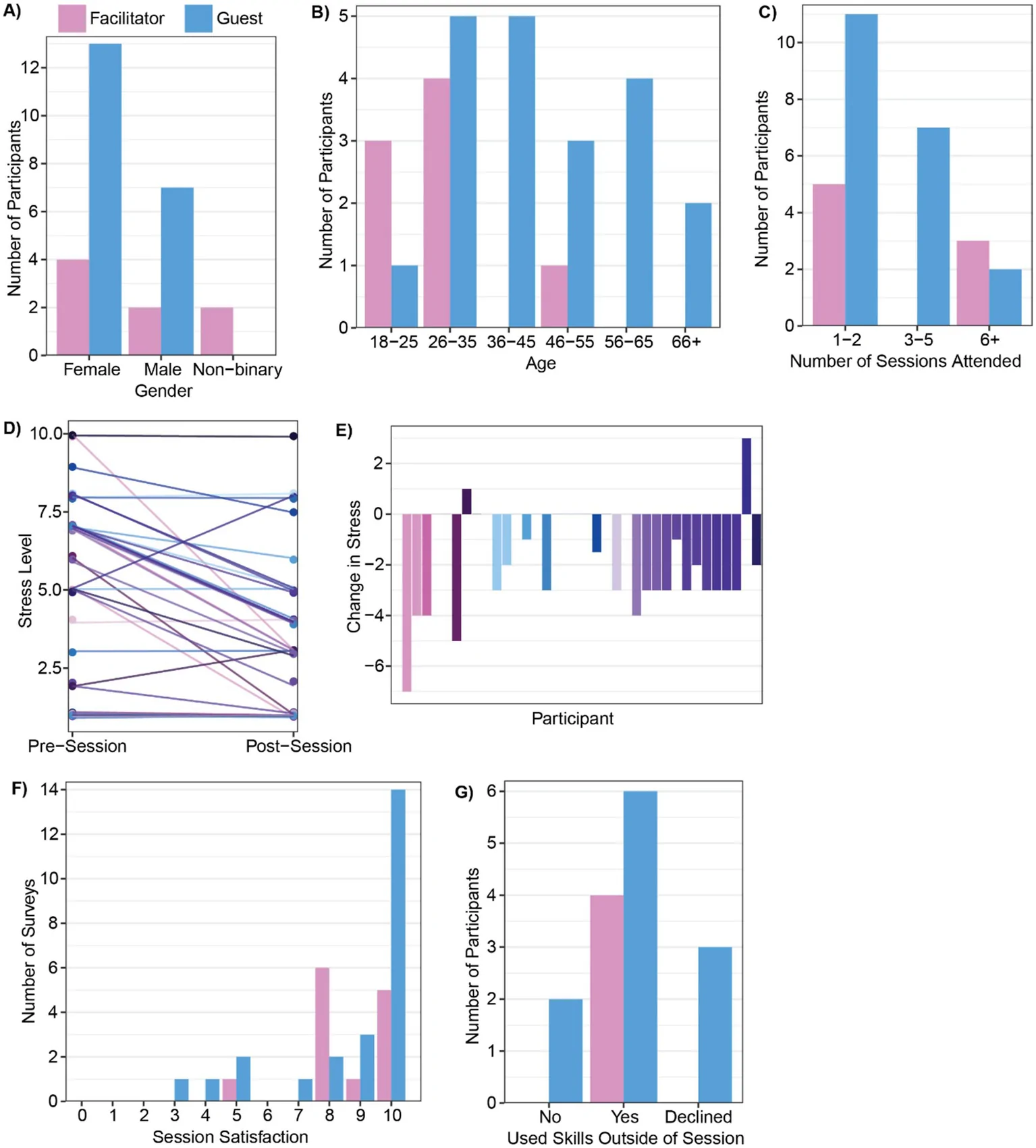
Art creation is associated with reduced stress. Unless otherwise specified, facilitators are colored pink and shelter guests are colored blue. **(A)** Quantification of participants by gender. **(B)** Quantification of participants by age bracket. **(C)** Total number of sessions attended by each participant at their most recent survey. **(D)** Self-reported stress levels at the beginning and at the end of the session. Each line represents a different survey, and lines are colored by participant. If a participant completed multiple surveys across different sessions, that participant will have multiple lines of the same color. **(E)** Change in participant stress levels between the pre-session survey and the post-session survey. Negative numbers represent a decrease in stress while positive numbers represent an increase in stress. Bars are colored as described in 1D). **(F)** Participants’ overall satisfaction at the end of the session. **(G)** Quantification of whether or not returning attendees used skills gained during the program in their everyday life.

To evaluate perceived impact, participants and facilitators completed pre and post-session surveys over a six-month period (Supplementary Figure S2). Respondents included a range of ages and genders, with the majority of respondents (13/20 guests and 4/8 facilitators) identifying as female (Figure 1A). While shelter guest ages were distributed across age groups (1/20 was 18–25, 5/20 were 26–35, 5/20 were 36–45, 3/20 were 46–55, 4/20 were 56–65, and 2/20 were 65+), facilitators were typically younger (3/8 were 18–25, 4/8 were 26–35, and 1/8 was 46–55), consistent with trainee involvement (Figure 1B). At the time of their most recent survey completion, nearly half of the participating shelter guests (9/20) had participated in more than two sessions, suggesting sustained engagement beyond initial curiosity (Figure 1C). Participants reported attending for various reasons including stress relief, enjoyment of the sessions, and convenience. Notably, even those who reported attending incidentally often described meaningful benefits post-session, indicating that perceived benefit was not limited to those purposefully seeking therapeutic gain.

Participants rated their stress levels before and after sessions using a 1–10 Likert-type scale. When pre and post-session stress levels (Figure 1D) and change in stress (Figure 1E) were plotted, the majority of respondents (22/36) reported a reduction in stress, with an average decrease of three points. While some reported no change and two reported increased stress, both individuals who experienced increased stress nonetheless reported moderate satisfaction and intention to return. Because this is such a subjective measure of stress, it is possible that these respondents did not consistently rate their stress pre and post-session. Nonetheless, this pattern suggests that even when immediate stress reduction did not occur, participants may have perceived other forms of value in the sessions.

Overall satisfaction was high, with a mean rating of 8.6/10 across respondents (Figure 1F). Nearly all participants indicated that they found the session helpful and would attend again. The single respondent who did not report benefit attributed this to preoccupation with an upcoming exam rather than dissatisfaction with the program itself, further underscoring the generally positive reception.

Importantly, beyond immediate stress reduction, participants identified coping strategies they drew on during and beyond the sessions (Figure 1G), including mindfulness, slowing down, relaxation, and improved artistic confidence. More than half of returning respondents (6/8) reported applying these strategies outside of sessions, suggesting that participants adapted art making as a portable coping resource.

The survey findings suggest that art creation was associated with immediate reductions in perceived stress for most participants, high satisfaction, and development of self-reported coping skills. Taken together, the survey findings corroborate facilitator observations and shelter staff reports, suggesting that structured art sessions may support perceived stress reduction, coping, and social connection within the shelter context.

## Discussion

In collaboration with a local homeless shelter, we developed an art creation program. During each session, participants were offered multiple art mediums and were encouraged to create art based on retrospection. At each session a new prompt was provided to encourage self-reflection. To analyze the efficacy and effects of the program, we utilized a mixed-methods approach that incorporated facilitator observations, interviews with shelter staff, and pre and post-session participant surveys. By integrating perspectives from multiple levels, we sought to assess not only individual-level outcomes but also broader environmental effects within the shelter.

Overall, the findings indicate strong acceptance and engagement with the program. High turnout and repeat participation suggest sustained interest beyond initial curiosity. While one interpretation of attendance is that it reflects limited recreational opportunities within the shelter, participation levels exceeded those typically observed in other externally facilitated programs.

The strong engagement observed across sessions is consistent with the core tenets of Self-Determination Theory (Deci and Ryan, 2000). The program was designed to support all three basic psychological needs: autonomy was preserved through participant choice of medium and voluntary attendance; competence was cultivated through skill acquisition and creative experimentation; and relatedness emerged organically through shared art-making and mutual disclosure. When people feel that their choices are respected, that they are growing in some meaningful way, and that they genuinely belong to a group, they are more likely to engage deeply and return consistently, and this is precisely what the attendance and repeat participation data reflect. Staff observations that the program’s informality and relational equality drove its unusually high uptake further support this reading. The trust built between facilitators and guests through co-participation rather than expert-directed instruction may have been a key mechanism through which the program reduced the hierarchical barriers that typically suppress engagement with externally facilitated programming in shelter settings.

The qualitative findings also align with Fredrickson (2001) Broaden-and-Build Theory. Participants consistently described experiences of enjoyment and connection during sessions, positive emotional states that open space for curiosity, creativity, and social risk-taking. This may help explain participants’ willingness to engage in identity exploration, emotional disclosure, and interpersonal vulnerability, behaviors that do not come easily in environments defined by instability and distrust, but which were observed consistently across sessions. Participants supported one another during vulnerable disclosures, celebrated achievements, and offered reassurance during stressful moments. Rather than remaining as individuals sharing physical space without deeper connection, participants functioned as a connected group whose interactions reinforced belonging. Notably, it became a reinforcing cycle. As trust increased, participants felt more comfortable sharing. As sharing deepened, connections were further strengthened. Importantly, positive emotions do not just feel good in the moment. When positive emotional states are experienced repeatedly, they build lasting internal resources. The spontaneous emergence of independent art use during moments of distress, and the shelter’s subsequent decision to integrate art materials into its everyday resources, suggests that these benefits extended beyond individual participants to shape the broader social environment of the shelter.

The quantitative findings both corroborate and extend the qualitative picture. The majority of participants reported immediate reductions in perceived stress following sessions, with an average decrease of three points on a ten-point scale, a meaningful shift for a single-session intervention that required no clinical infrastructure. Notably, even among the two participants who reported increased stress, satisfaction remained moderate and both indicated intention to return, suggesting that the value of the sessions was not reducible to immediate stress relief alone. This is consistent with the qualitative themes: participants described the sessions as meaningful spaces for connection, reflection, and identity expression, benefits that may not always manifest as a reduction in acute stress but are nonetheless real and significant. The high mean satisfaction rating of 8.6 out of 10, sustained across a diverse group of participants with varying attendance histories, further suggests that the program’s appeal was broad rather than limited to those who were already predisposed to benefit. Perhaps most striking is the finding that more than half of returning participants reported applying coping skills: mindfulness, slowing down, and intentional reflection outside of the sessions. This transfer of skills beyond the structured program context is not a trivial finding. It suggests that the sessions functioned not merely as a temporary reprieve from stress but as a setting in which participants developed and internalized portable coping strategies, a pattern consistent with both the SDT and Broaden-and-Build frameworks outlined earlier. Together, these findings suggest that even a brief, low-resource creative program can produce meaningful and measurable psychological benefits across multiple levels, from immediate stress relief to the development of coping strategies that participants carried into their daily lives.

Our findings both align with and extend prior work on arts-based programming for people experiencing homelessness. The high engagement we observed is notable given documented barriers to service acceptance in this population: reviews of health and social interventions for people experiencing homelessness consistently identify trust and personal safety as determinants of acceptability (Magwood et al., 2019) and institutional distrust as a barrier to service use (Kerman et al., 2019). Our experience suggests that informal, co-participatory programming may be one way to work within these documented barriers. The stress reduction participants reported is consistent with experimental evidence that art making lowers physiological markers of stress (Kaimal et al., 2016) and with the broader open studio literature (Finkel and Bat Or, 2020). It builds upon Schwan et al.' (2018) finding that young people experiencing homelessness identify art creation as one of their most valued self-care practices, suggesting this is similar for adults in congregate shelter settings as well. Similarly, the themes of identity expression, emotional processing, and social connection echo those identified in Murphy and Alexander (2020) meta-synthesis of arts-based programming for adults experiencing homelessness, as well as earlier work with homeless women and youth (Prescott et al., 2008; Stokrocki et al., 2004). What prior work has largely not captured and where our findings add something new is evidence of change at the level of the shelter itself. Additionally, our work provides concrete implementation and engagement data from an adult congregate setting.

This study makes three contributions. First, to our knowledge, it is among the first mixed-methods evaluations of a non-clinical art creation program for adults in congregate shelter housing that explicitly assesses implementation and engagement, a gap identified in the literature. Second, it offers a documented, replicable model that does not depend on licensed art therapists, responding directly to calls to reframe creative programming around the psychosocial and financial realities of homelessness services (Griffith et al., 2015; Regev and Cohen-Yatziv, 2018). Third, it extends the evidence base beyond individual-level outcomes. The integration of art materials into the shelter’s everyday resources and the spontaneous use of art during distress suggest that brief, recurring creative programming can shift the culture of a shelter, not just the experience of individual participants.

These findings carry several practical implications for shelters, community organizations, and program designers. The program’s low cost and minimal resource requirements suggest that art creation programs are scalable: requiring little more than basic art supplies, a communal space, and willing facilitators. Unlike formal mental health services, this model does not depend on licensed clinicians or referral pathways, making it embeddable within existing shelter infrastructure without significant financial investment. The relational design of the program also appeared critical to its success. Co-participation of facilitators as peers rather than authority figures seemed to drive engagement in ways that more traditional service-delivery models may not achieve, particularly among populations with histories of institutional distrust. Finally, the spontaneous emergence of art as a coping tool beyond the structured sessions suggests that even brief, recurring programs may contribute to broader cultural shifts within shelter environments, with potential for creative expression to become an integrated part of daily life rather than a standalone intervention.

Several limitations should be considered when interpreting these findings. First, this study was conducted in a single shelter with a relatively small sample size, limiting generalizability to other shelter or community settings. Second participation in surveys and interviews was voluntary, potentially introducing response bias. Individuals who chose to provide feedback may have been more engaged or more positively inclined towards the program, potentially inflating perceived benefits. Additionally, all quantitative data was measured using self-reported data that had not been psychometrically validated, which increases the risk of bias and variability in interpretation of rating scales. Longer-term follow up was not conducted to evaluate the durability of the observed effects. Thirdly, some of the facilitators were also implementers of the program and study. This inherently introduces bias. Finally, although staff perceptions suggest broader cultural shifts within the shelter, these were subjective observations and may not accurately reflect the effects of the program. Additionally, since we did not collect staff demographics to protect their anonymity, we are unable to characterize the staff interviewees.

## Conclusion

This study suggests that a low-cost art making program may support perceived stress reduction, self-directed coping, and social connection among individuals residing in a homeless shelter. Using a multi-method evaluation approach, we found that participants not only reported immediate psychological benefits but also developed coping skills that extended beyond the sessions.

Crucially, the program’s impact extended beyond individual outcomes to influence the broader shelter culture. Art became embedded as an accessible tool for emotional expression and regulation, evidenced by spontaneous requests for materials and increased use during moments of distress. These findings suggest that even simple, low-resource programs may influence shelter routines and norms when they are accessible, voluntary, and responsive to participants’ interests.

Taken together, this work offers a practical model: a creative program that requires no licensed clinicians, minimal materials, and modest facilitator time, yet achieved sustained engagement in a population where engagement is notoriously difficult. Notably, the elements that appeared most critical to the program’s success were also the simplest to implement: voluntary attendance, choice of medium, and facilitators who create alongside participants rather than direct them.

Although this program was implemented in a single urban shelter in the United States, it does not need to be specific to this setting. Day programs, transitional housing, drop-in centers, warming/cooling shelters, and other congregate services face similar constraints, namely, limited budgets, limited clinical staffing, and populations with understandable distrust of formal services. The model described here could reasonably be adapted to these contexts, including internationally, where access to credentialed art therapists is often even more limited.

Future research should evaluate this approach with larger samples, controlled designs, and psychometrically validated measures of stress and wellbeing. Longitudinal follow-up would clarify whether the coping skills participants reported persist over time, and multi-site implementation studies would test whether the model transfers across shelter types and organizational cultures. Comparative work examining different facilitator backgrounds (i.e., psychology trainees, shelter staff, and community volunteers) could help identify the minimum training needed to deliver the program safely. In the meantime, our findings indicate that community-based creative programming may meaningfully contribute to resilience, social cohesion, and emotional wellbeing in shelter settings.

## Data Availability

The raw data supporting the conclusions of this article will be made available by the authors, without undue reservation.
