# Supplementary material for: Perceived impact of a homeless shelter-based art program on stress, social connection, and emotional regulation: a mixed-methods pilot study
Source: Front Psychol. 2026 Aug 26;17:1825416. doi: 10.3389/fpsyg.2026.1825416 (PMC13553932; doi:10.3389/fpsyg.2026.1825416)
Supplement: Supplementary file 1 [file Supplementary_File_1.pdf]

South Park Inn staff/administration interview

1. How long have you been working at South Park Inn?
2. Have you participated in any of the art sessions?
3. If you have participated, what was your impression of them?
4. Do you know of any other staff who have participated in the art sessions?
5. If you know any staff who have participated, what was their impression of them?
6. How do you think the shelter guests feel about the art sessions?
7. Do you feel like the art sessions are offering any benefit to the shelter and the guests?
8. Have you noticed any changes in the shelter guests who participate in the art sessions?
9. Do you feel like the art sessions are helping their stress levels?
10. Do you feel like the art sessions are helping them cope with stressors?
11. Is there anything else regarding the art sessions you wish to share?
