# Supplementary material for: Perceived impact of a homeless shelter-based art program on stress, social connection, and emotional regulation: a mixed-methods pilot study
Source: Front Psychol. 2026 Aug 26;17:1825416. doi: 10.3389/fpsyg.2026.1825416 (PMC13553932; doi:10.3389/fpsyg.2026.1825416)
Supplement: Supplementary file 2 [file Supplementary_File_2.pdf]

Pre-group survey

1. Select your age below.
   18 – 25 26 – 35 36 – 45 46 – 55 56 – 65 65+

2. Select your gender below.
   Male Female Non-binary

3. Have you attended this art group previously?
   Yes No

4. If you have attended before, how many times have you attended?
   1-2 times 3-5 times 6+ times

5. If you have attended this art group before, what have you learned from this art group?

6. If you have attended before, have you used what you learned in this art group outside the session?
   Yes No

7. If you have used what you learned in this art group outside the sessions, how have you used what you learned?

8. On a scale of 1, being "not at all," through 10, being "extremely," how severe is your stress at this time? Please fill in one circle for your response.

Not at all				Moderately stressed					Extremely stressed
1	2	3	4	5	6	7	8	9	10
○	○	○	○	○	○	○	○	○	○

9. Why are you attending today's session? Please write your answer below.

Post-group survey

1. On a scale of 1, being "not at all," through 10, being "extremely," how severe is your stress at this time? Please fill in one circle for your response.

Not at all				Moderately stressed					Extremely stressed
1	2	3	4	5	6	7	8	9	10
○	○	○	○	○	○	○	○	○	○

2. On a scale of 1, being "not at all," through 10, being "highly," how satisfied were you with this group therapy activity? Please fill in one circle for your response.

Not at all				Somewhat satisfied					Highly satisfied
1	2	3	4	5	6	7	8	9	10
○	○	○	○	○	○	○	○	○	○

3. What activity did you choose? Please write your answer below.

4. What activity would you like to do during the next session? Please write your answer below.

5. Did you think today's session was helpful?
   Yes No

6. Would you attend this group again?
   Yes No

7. What did you like about today's session? Please write your answer below.

8. Would you recommend these sessions to another resident?
   Yes No

9. What would you change about these sessions to make them more helpful? Please write your answer below.
